# Combined Evaluation of FDG-PET and MRI Improves Detection and Differentiation of Dementia

**DOI:** 10.1371/journal.pone.0018111

**Published:** 2011-03-23

**Authors:** Juergen Dukart, Karsten Mueller, Annette Horstmann, Henryk Barthel, Harald E. Möller, Arno Villringer, Osama Sabri, Matthias L. Schroeter

**Affiliations:** 1 Max Planck Institute for Human Cognitive and Brain Sciences, Leipzig, Germany; 2 Day Clinic of Cognitive Neurology, University of Leipzig, Leipzig, Germany; 3 Department of Nuclear Medicine, University of Leipzig, Leipzig, Germany; University of California, San Francisco, United States of America

## Abstract

**Introduction:**

Various biomarkers have been reported in recent literature regarding imaging abnormalities in different types of dementia. These biomarkers have helped to significantly improve early detection and also differentiation of various dementia syndromes. In this study, we systematically applied whole-brain and region-of-interest (ROI) based support vector machine classification separately and on combined information from different imaging modalities to improve the detection and differentiation of different types of dementia.

**Methods:**

Patients with clinically diagnosed Alzheimer's disease (AD: n = 21), with frontotemporal lobar degeneration (FTLD: n = 14) and control subjects (n = 13) underwent both [F18]fluorodeoxyglucose positron emission tomography (FDG-PET) scanning and magnetic resonance imaging (MRI), together with clinical and behavioral assessment. FDG-PET and MRI data were commonly processed to get a precise overlap of all regions in both modalities. Support vector machine classification was applied with varying parameters separately for both modalities and to combined information obtained from MR and FDG-PET images. ROIs were extracted from comprehensive systematic and quantitative meta-analyses investigating both disorders.

**Results:**

Using single-modality whole-brain and ROI information FDG-PET provided highest accuracy rates for both, detection and differentiation of AD and FTLD compared to structural information from MRI. The ROI-based multimodal classification, combining FDG-PET and MRI information, was highly superior to the unimodal approach and to the whole-brain pattern classification. With this method, accuracy rate of up to 92% for the differentiation of the three groups and an accuracy of 94% for the differentiation of AD and FTLD patients was obtained.

**Conclusion:**

Accuracy rate obtained using combined information from both imaging modalities is the highest reported up to now for differentiation of both types of dementia. Our results indicate a substantial gain in accuracy using combined FDG-PET and MRI information and suggest the incorporation of such approaches to clinical diagnosis and to differential diagnostic procedures of neurodegenerative disorders.

## Introduction

In recent research, various biomarkers have been reported to differentiate between early stages of dementia and healthy control subjects or between different types of neurodegenerative disorders, suggesting an integration of these would improve diagnostic accuracy of dementia [1]–[9].

For the detection of dementia, accuracy rates significantly above 90% have recently been reported using univariate and multivariate statistical approaches in magnetic resonance imaging (MRI) and [F18]fluorodeoxyglucose positron emission tomography (FDG-PET) [1],[10]–[14]. However, the differentiation of the two most common types of dementia, namely Alzheimer's disease (AD) and frontotemporal lobar degeneration (FTLD), is still problematic. For this differentiation, accuracy rates ranging between 84 and 89% are still in need of improvement, especially due to a substantially lower sensitivity compared with specificity of actual methods [10],[12],[15]. Nevertheless, the use of biomarkers has significantly helped to improve diagnostic accuracy compared with diagnoses based solely on clinical and neuropsychological evaluation [16],[17]. For these reasons, recent studies have suggested to incorporate imaging findings into criteria for diagnosis of dementia [17],[18].

For AD patients imaging studies have shown reduced glucose consumption mainly in parietotemporal and posterior cingulate cortices [9],[20],[21] and structural changes in the hippocampus and entorhinal area relative to healthy controls [9],[21],[22]. In FTLD patients, atrophy and reduced metabolic rate for glucose have been reported to be predominately located in the medial thalamus, amygdala and in frontotemporal and anterior cingulate cortices [4],[8],[20],[21],[23].

For multivariate differentiation of different types of dementia support vector machine classification (SVM) is used based on whole-brain voxel information [10] or most frequently on ROI values [6],[12]–[13],[24]–[26]. A major problem of the ROI-based approach is the limited generalizability of the trained classifier, because the ROIs are selected based on features showing a between-group differentiation in the same groups in a univariate analysis. Although ROIs selected with this method provide a good discrimination between groups used in these specific studies, they might show significantly reduced discrimination power when applied to new data sets. This could be the case if the selected regions just detect differences between groups, which are not necessarily attributed to the specific neurodegenerative disorder. Furthermore, AD and FTLD patients have been shown to develop a differential regional pattern of glucose hypometabolism and atrophy [21],[27]. However, the previously proposed approaches only used single modality information for the classification algorithms loosing this way the differential information which various biomarkers might provide for a better detection and differentiation of dementia syndromes.

Here, we apply SVM as the most frequently used multivariate approach to evaluate its contribution for detection and differentiation of dementia in multimodal imaging. To increase the validity of our method we apply SVM classification on data extracted from ROIs based on disorder-specific metabolic reductions and atrophy reported in comprehensive meta-analyses investigating AD and FTLD. This method allows a better generalization of our classification algorithms to other clinical centers and ensures that only disorder-specific changes are used for SVM based discrimination. We hypothesize that common use of different imaging modalities might substantially improve early detection and differentiation of dementia.

## Methods

### Ethics Statement

The research protocol was approved by the Ethics Committee of the University of Leipzig, and was in accordance with the latest version of the Declaration of Helsinki. Informed consent was obtained from all subjects.

### Subjects

We analyzed FDG-PET and T1-weighted MRI data of 21 patients (Table 1) with an early stage of probable AD, 14 patients with an early stage of FTLD and 13 control subjects. Patients were recruited from the Day Clinic of Cognitive Neurology at the University of Leipzig. Probable AD was diagnosed according to NINCDS-ADRDA criteria [28]. Although all AD subjects also fulfilled the revised NINCDS-ADRDA criteria suggested by Dubois et al. [17] the fulfillment of the original McKhann criteria was sufficient for the inclusion into the study. Diagnosis of FTLD was based on criteria suggested by Neary et al. [29]. The control group included subjects who visited the Day Clinic with subjective cognitive complaints, which were not objectively confirmed by a comprehensive neuropsychological and clinical evaluation. FDG-PET and MRI for these subjects was conducted for diagnostic reasons within the clinical assessment. This control group was chosen because, in clinical practice, it is crucial to discriminate between these subjects showing a normal age-related decrease in cognitive performance and patients with an early stage of dementia. Patients were excluded if structural imaging revealed lesions due to stroke, traumatic head injury, brain tumor or inflammatory diseases.

**Table 1:** Subject group characteristics.

	Controls	AD	FTLD	ANOVA (df,F,P)
Number	13	21	14	–
Male/Female	7/6	9/12	7/7	–
Age (years)	53.9±6.0	61.1±6.7	60.8±6.4	2, 5.76, 0.006
CDR (score)	0.23±0.26	0.71±0.25	0.82±0.42	2, 13.93, 0.000
MMSE (score)	n.a.	23.2±3.9	24.4±4.2	–
Education (years)	12.3±3.1	10.7±3.1	11.6±3.8	2,1.02,0.368

Mean ± standard deviation. AD Alzheimer's disease, ANOVA analysis of variance, CDR Clinical Dementia Rating Scale, FTLD frontotemporal lobar degeneration, MMSE Mini Mental State Examination, n.a. not available.

### Data acquisition

#### MRI data

For each subject, a high-resolution T1-weighted MRI scan was obtained, consisting of 128 sagittal slices adjusted to AC-PC line and a with slice thickness of 1.5 mm and pixel size of 1×1 mm^2^. MRI was performed on two different 3T scanners (MedSpec 30/100, Bruker Biospin, Ettlingen Germany and Magnetom Trio, Siemens, Erlangen, Germany) using two different T1-weighted sequences (MDEFT or MP-RAGE with TR = 1300 ms, TI = 650 ms, TE = 3.93 ms or TE = 10 ms; FOV 25×25 cm^2^; matrix  =  256×256 voxels). On the MedSpec scanner, only the MDEFT-sequence and on the Magnetom Trio scanner, either MDEFT or MP-RAGE sequences were used. The distribution of scanner types and sequences used to obtain the MRI data was random across subjects and did not differ significantly in its distribution between the groups nor for scanner type nor for sequence.

#### PET data

Each subject also underwent FDG-PET imaging either a few a weeks before or after the MRI scan. All PET data were acquired on a Siemens ECAT EXACT HR+ scanner (CTI/Siemens, Knoxville, TN, USA) under a standard resting condition in 2-dimensional (2D) mode. The 2D acquisition mode was used because it allows a better quantification of the PET data due to lower scatter radiation. Sixty-three slices were simultaneously collected with an axial resolution of 5 mm full width at half maximum (FWHM) and in-plane resolution of 4.6 mm. After correction for attenuation, scatter, decay and scanner-specific dead-time, images were reconstructed by filtered back-projection using a Hann-filter of 4.9 mm FWHM. The 63 transaxial slices obtained had a matrix of 128×128 voxels with an edge length of 2.45 mm.

### Image processing and statistical analysis

**Figure 1:**
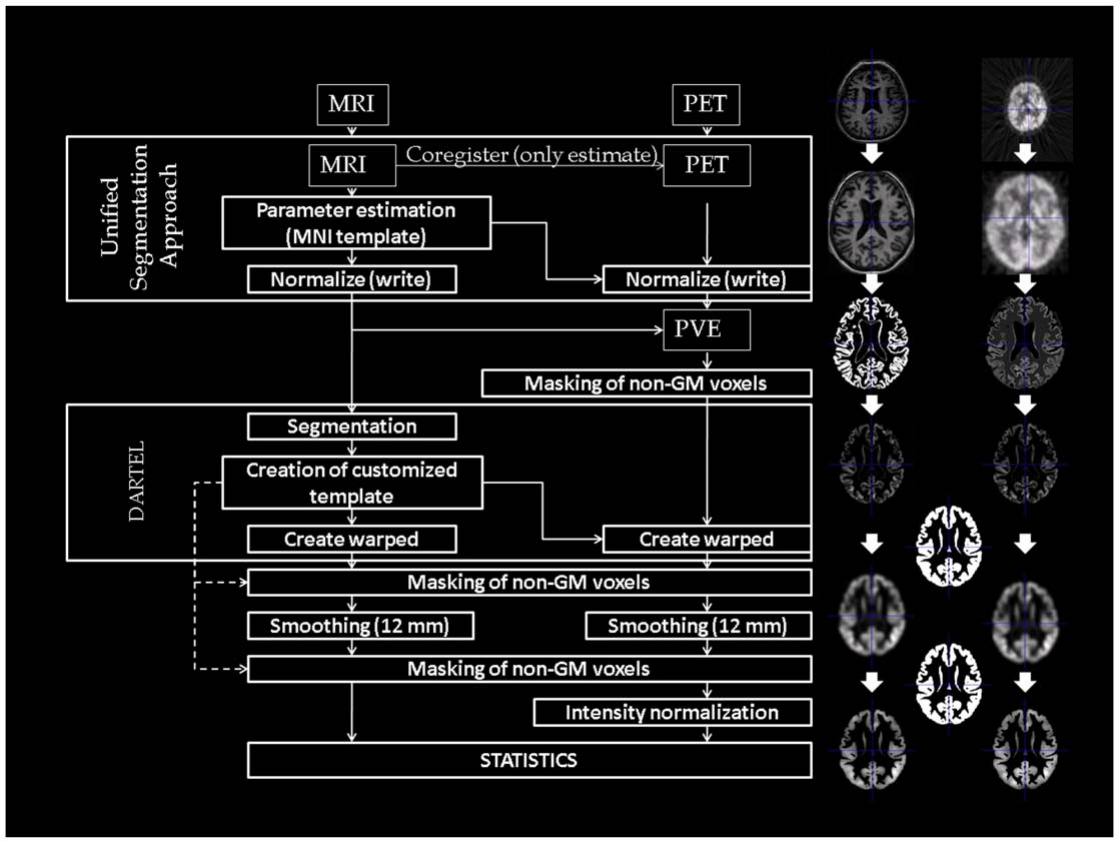
Schematic representation of the procedure for FDG-PET and MRI data handling and processing steps. FDG-PET [F18]fluorodeoxyglucose positron emission tomography, MRI magnetic resonance imaging, MNI Montreal Neurological Institute, PVE partial volume effect correction.

The procedure described below has been specifically designed for this study, aiming at a most accurate co-processing of FDG-PET and MRI data to obtain a more precise between subject anatomical overlap (Figure 1). All image-processing steps were carried out using the SPM5 software package (Statistical Parametric Mapping software: http://www.fil.ion.ucl.ac.uk/spm/) implemented in Matlab 7.7 (MathWorks Inc., Sherborn, MA). SVM classification was conducted with the LIBSVM software [30] using the Matlab interface.

#### MR images

The MR images were first interpolated to get an isotropic resolution of 1×1×1 mm^3^. The resultant MR images were coregistered on their respective FDG-PET images and bias corrected for inhomogeneity artifacts using the Unified Segmentation Approach described in detail by Ashburner and Friston [31]. This specific method performs a better coregistration of images from different modalities and allows a more accurate segmentation due to the bias correction. A further reason to use this approach was that the straightforward coregistration implemented in the PVElab software described later sometimes failed. We used this software for automatic partial volume correction of the FDG-PET images. The coregistered MR images were processed using the DARTEL (Diffeomorphic Anatomical Registration Through Exponentiated Lie algebra) approach [32] to enable a more accurate spatial normalization. This approach registers all gray matter (GM) and white matter (WM) images to an averaged-size template created from all subjects used in this study and modulates the images to preserve the total amount of signal from each region in the images. Subsequently, the images were smoothed using a Gaussian kernel of 12 mm FWHM. This smoothing factor, although higher then usual MR kernels, was selected based on extensive tests, because it allows the optimal coevaluation with lower resolution FDG-PET images.

#### FDG-PET data

Within the common registration with MRI data using the Unified Segmentation Approach described above, PET images were interpolated to the same voxel size as the MR images, namely 1×1×1 mm^3^. This processing does not introduce any additional noise into the PET images. However, in our experience, it substantially improves the subsequent partial volume effect (PVE) correction of voxels representing GM intensities using the modified Müller-Gärtner method [33],[34]. Due to the interpolation, they are exactly overlaid with the MR tissue class images of the same subject obtained from the segmentation step in the PVE approach. Thus, the within-voxel correction is done only for those voxels directly overlaying the GM structures in the MR images. Instead of smoothing the MR data to the resolution of PET data and thus loosing the exact quantitative and qualitative information of GM distribution, which is usually done in the PVE correction, the interpolation of FDG-PET preserves this information. This allows a more accurate correction of atrophy effects onto glucose utilization. The subsequent PVE correction including all image processing steps was done by using the automatic algorithm implemented in the PVElab software package [35]. Because the modified Müller-Gärtner method sets all WM voxel values to the mean WM intensity value, these regions do not contain any further valuable regional information after the PVE correction. For this reason, all voxels belonging to WM were masked using the ImCalc function in the SPM5 software package by filtering this specific intensity in the whole image. After the PVE correction, the DARTEL flow fields calculated from the MR images were applied to their respective PET images to obtain an anatomically exact overlap between GM and PET images of all subjects with modulation to preserve the total amount of signal from each region. In the same way as the MR data, the PET data were smoothed by a Gaussian kernel of 12 mm FWHM. Finally, the FDG-PET data were intensity normalized using cerebellar ROIs to account for individual differences in global PET measures. This region has been shown to be least affected in mild to moderate stages of AD [36]. Additionally, normalization to this region improves the statistical discrimination between dementia patients and control subjects in comparison to other regions reported in the literature [37]–[39].

#### Masking

The MR and PET images obtained as described above were masked to avoid contamination by misclassified voxels. Voxels lying between WM and ventricular cerebrospinal fluid tend to be misclassified as GM voxels due to their similar intensity. The mask was obtained after extensive testing by excluding all voxels in the first and the last template created by the DARTEL approach with a probability of below 0.2 for belonging to GM and including only the voxels that exceed this threshold in both templates. This mask was applied twice: firstly prior to smoothing to avoid misclassification, and secondly, after the smoothing to avoid big edge effects. WM images were exclusively masked using the same mask to avoid overlaps between GM and WM voxels due to smoothing. The masked images were used for the subsequent SVM analysis of the data.

#### ROI extraction

**Figure 2:**
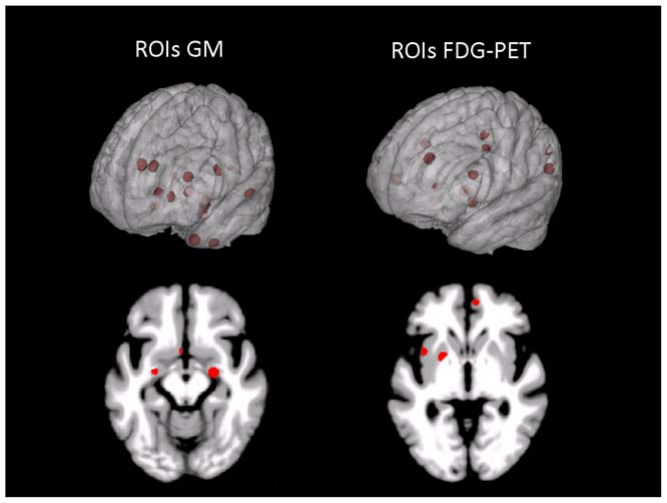
Regions of interest extracted from gray matter (left) and FDG-PET (right) data for AD and FTLD patients and used for support vector machine classification projected onto a glass brain (top) and onto an axial slice (bottom). AD Alzheimer's disease, FDG-PET [F18]fluorodeoxyglucose positron emission tomography, FTLD frontotemporal lobar degeneration, GM gray matter, ROIs regions of interest.

ROI coordinates (Table 2) were extracted from two comprehensive, systematic and quantitative meta-analyses investigating biomarkers of AD and FTLD in MR and FDG-PET images. The meta-analyses included a total number of 1618 patients (AD/FTLD: 1351/267) and 1448 healthy control subjects (1097/351) [8],[9]. These meta-analyses extracted the prototypical networks of AD and FTLD by applying what is currently the most sophisticated and best-validated of coordinate-based voxel-wise meta-analyses, anatomical likelihood estimate. In the FTLD meta-analyses [8] only coordinates which are common to all subgroups of FTLD patients were used. In total, 10 regions from MRI and 6 regions from FDG-PET were used from the FTLD meta-analysis. From AD meta-analysis, 6 regions were used from both, MRI and FDG-PET. The AD meta-analysis also identified one additional region in the fornix which was differentiating between early-onset AD patients (age<65 years) and control subjects but not between late-onset AD patients and control subjects. This region was not included into the ROI-based classification to avoid a discrimination bias towards early-onset AD patients. Although unequal numbers of ROIs were used from both imaging modalities, this number is also a highly important information as it also provides a measurement for the amount of changes present in a specific modality. Because the coordinates in both meta-analyses were reported in the Talairach space, they were transformed to MNI space according to a formula proposed by Matthew Brett (published on the Internet: http://www.mrccbu.cam.ac.uk/Imaging/Common/mnispace.shtml). DARTEL preprocessed data are registered to an averaged size template created from all subjects in this study. To transform these data to the MNI space we normalized them to an a priori MNI template in SPM by using affine-only spatial normalization. Due to the affine-only transformation, our images still differed in shape from the MNI template, so some reported coordinates were slightly outside of the anatomic regions in our imaging data. In this case, the center coordinates for the ROIs were moved slightly towards the closest point of the corresponding anatomical region reported in the meta-analyses. ROIs were selected using the 3D fill tool in the MRIcron software package (http://www.sph.sc.edu/comd/rorden/mricron). Separate ROI masks were created for MR and FDG-PET images based on the origin of the peak values reported in the meta-analyses and using all regions reported for AD and FTLD in a single mask for each modality. Each ROI was restricted to a sphere with a radius of 5 mm around the reported coordinate (Figure 2). Additionally, to increase the signal-to-noise ratio, all zero voxels and edge voxels with an intensity deviation of 13 intensity units in the MRIcron 3D fill tool were excluded from the ROI. The edge voxel restriction excludes all voxels at the edge of the smoothed GM structures within the sphere. These voxels carry much less information due to their further distance from the GM structures in the unsmoothed data and so decrease the signal-to-noise ratio in the corresponding ROI.

**Table 2:** Coordinates of ROIs used for SVM classification.

FTLD vs. Controls					
FDG-PET	BA	Lat	x	y	z
Pregenual anterior cingulate gyrus	24/32	L	−5	34	21
Lentiform nucleus; Caudate head		L	−20	3	−3
Medial thalamus		L	−2	−19	6
Anterior insula	15/16	L	−47	10	−7
Anterior medial frontal cortex	10	R	1	54	0
Amygdala		R	25	−2	−25

MRI	BA	Lat	x	y	z
Anterior medial frontal cortex	9	L	−5	49	25
Pregenual anterior cingulate gyrus	24/32	L	−5	34	21
Inferior frontal gyrus, pars opercularis	44	L	−54	11	20
Lentiform nucleus; Caudate head		L	−20	3	−3
Anterior insula	15/16	L	−47	10	−7
Subcallosal/septal area	25	L	−2	10	−10
Amygdala		R	25	−2	−25
Amygdala; Ento-and perirhinal cortex		L	−26	−5	−25
Temporal pole	38	L	−29	11	−43
Temporal pole	38	L	−47	8	−44

AD vs. Controls					
FDG-PET	BA	Lat	x	y	z
Angular gyrus	39	L	−38	−68	37
Angular gyrus	39	R	43	−68	33
Posterior superior temporal sulcus	21/22				
Anterior medial frontal cortex	9/10	R	1	31	31
Pregenual anterior cingulate gyrus	32				
Inferior precuneus	31	R	1	−36	27
Dorsal posterior cingulate cortex	23				
Posterior superior temporal sulcus	21/22	L	−51	−61	23
Middle inferior temporal sulcus	20/21	R	59	−31	−23

MRI	BA	Lat	x	y	z
Posterior insula	13	L	−38	−25	15
Medial thalamus		L	−5	−13	3
Hippocampal body/tail		R	31	−38	−6
Middle temporal gyrus/superior temporal sulcus	21/22	L	−63	−21	−5
Amygdala, anterior hippocampal formation, uncus, (trans-) entorhinal area	28/34	R	25	−8	−18
Amygdala, anterior hippocampal formation, uncus, (trans-) entorhinal area	28/34	L	−26	−8	−18

Coordinates are in MNI space (L left, R right). AD Alzheimer's disease, BA Brodmann area, FDG-PET [18F]fluorodeoxyglucose positron emission tomography, MRI magnetic resonance imaging, ROI region-of-interest, SVM support vector machine.

#### SVM

Multivariate pattern classification, as described in Klöppel et al. [10], was performed with a linear kernel by identifying a separating hyperplane that maximizes the distance between different clinical groups based on whole-brain or ROIs information. The cross-validation of the trained SVM was performed by using the leave-one-out method. This procedure iteratively leaves out the information of each subject and trains the model on the remaining subjects for subsequent class assignation of the person that was not included in the training procedure. This validation method enables the generalization of the trained SVM to data that have never been presented to the SVM algorithms previously. The reported accuracy is the percentage of subjects correctly assigned to the clinical diagnosis. Usually SVM classification is performed without smoothing of the data, because single voxels are assumed to contain information, for example, for prediction of future action based on functional MR images. However, in neurodegenerative disorders single voxels are unlikely to contain generalizable information due to a limited across-subject registration of MR and FDG-PET images. Although SVM classification based on unsmoothed data has been shown to differentiate reasonably between different groups (Klöppel et al., 2007), an additional smoothing should make this approach more reliable and generalizable to new data. To control for the effect of smoothing we ran the same whole-brain classification twice for GM, PET and for integration of GM and PET in the same vector with and without smoothing.

We performed the whole-brain SVM classification using GM, WM or FDG-PET images separately and by combining information from different modalities. For the SVM classification, all data of a subject are transformed into a vector, with information of an additional modality simply attached by extending the vector. Additionally, we repeated the whole-brain SVM classification by adding MR to FDG-PET information combining both modalities in a single image. ROI-based SVM classification was performed on data extracted from smoothed images separately for GM and FDG-PET images and also by integrating information from both modalities in a single vector. In order to reduce the number of voxels in the ROI-based classification, only nonzero voxels were included in the vector. This was done because otherwise the whole-brain SVM classification is a highly memory-consuming approach. To ensure that our classification results were not based on factors randomly discriminating between groups, we reran the whole-brain and ROI-based classification for comparison 30 times by randomly assigning all subjects to the three groups independently from the clinical diagnosis and calculating the classification accuracy by using the leave-one-out procedure described above.

#### Statistical analysis

Group comparisons for age, education and CDR (Clinical Dementia Rating Scale) [40] were performed by conducting ANOVAs (analyses of variance). If an ANOVA revealed a significant between-group effect, a Bonferroni t-test was calculated with a significance threshold of p<05 (corrected for multiple comparisons, two-tailed). MMSE (Mini Mental State Examination) [41], was only present for 20 patients with AD and 11 patients with FTLD. MMSE scores of these two groups were compared to each other using an independent samples t-test. Group differences regarding sex were evaluated using a chi-square test for independent samples. The statistical analysis was performed with the commercial software package SPSS 17.0 (http://www.spss.com/statistics/).

## Results

### Clinical characteristics

The chi-square test for independent samples did not reveal any statistical differences in sex between the groups [χ^2^(2) = 0.42;p = 0.809]. The three groups did not differ in education (Table 1). CDR scores differed significantly in the three groups. The post-hoc test revealed no differences in the mean CDR scores between both groups of dementia patients [t(33) = 0.94;p = 0.977]. As expected, both early AD [t(32) = 5.36;p<0.001] and early FTLD [t(25) = 4.35;p<0.001] had significantly higher CDR scores compared to the control subjects. MMSE scores also did not differ between both groups of dementia patients indicating a similar severity of dementia syndrome [t(29) = −81;p = 0.95]. The ANOVA also revealed a significant group difference in age. The two groups of dementia patients did not differ significantly in age [t(33) = 0.16;p = 1.0]. There was a minor but significant difference between AD patients and controls [t(32) = 3.18;p = 008] and FTLD patients and controls [t(25) = 2.86; p = 024].

### SVM – Whole-brain analysis

**Figure 3:**
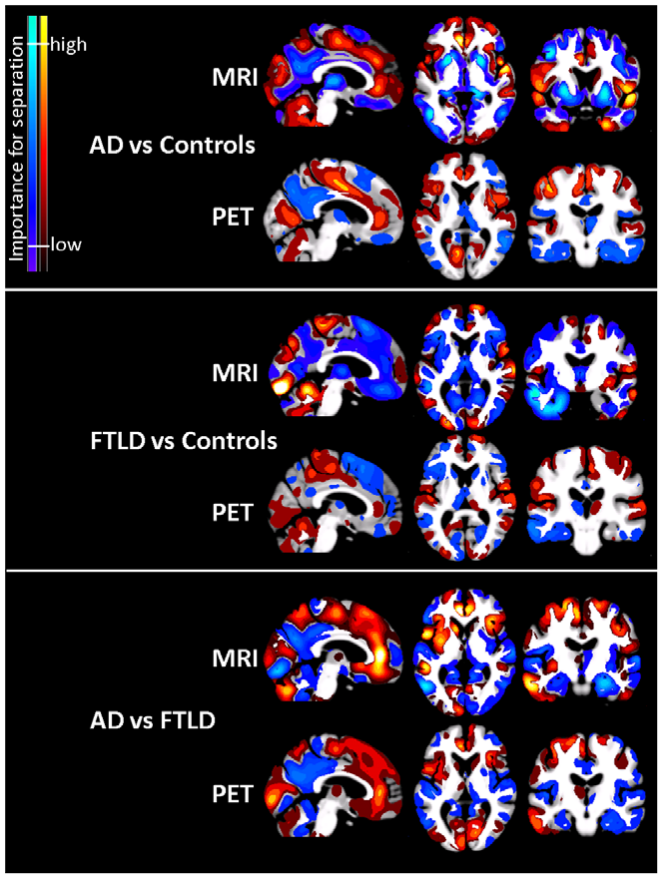
Weights of voxels most relevant for classification of both groups of patients and control subjects in FDG-PET and MRI after SVM training. Weights are relative, and have no applicable units. AD and FTLD vs Controls: Blue and light blue indicate decreased gray matter intensity (upper row) or reduced metabolic rate (lower row) that increase the likelihood of classification into a dementia group. Red and yellow indicate the opposite. AD vs FTLD: Blue and light blue indicate decreased gray matter intensity (upper row) or reduced metabolic rate (lower row) that increase the likelihood of classification into the AD group. Red and yellow indicate the opposite. Regions with bright colors (yellow and light blue) have a higher importance for separation than regions with dark colors (blue and red). AD Alzheimer's disease, FTLD frontotemporal lobar degeneration. MRI magnetic resonance imaging, PET positron emission tomography.

Multivariate classification of the data using SVM at the whole-brain level revealed the best discrimination accuracy for all three groups using FDG-PET, with 81% (chance level 33%), in comparison to GM and WM information, with lowest accuracy using WM information on its own (Table 3). The combination of metabolism and GM values in a single image revealed a similar accuracy for differentiation of the three groups, with higher accuracy for differentiation between both types of dementia, however, with slightly lower discrimination between dementia patients and control subjects. Whole-brain SVM classification for the three groups without smoothing revealed lower accuracy rates in all classifications in comparison to differentiation based on smoothed images. The accuracy increase due to smoothing ranged between 2 (GM) and 6% (FDG-PET). Figure 3 displays regions that were most influential in making binary classification between the AD, FTLD and control subjects based on smoothed whole-brain information.

**Table 3:** Accuracy rates for whole-brain and ROI-based SVM classification for FDG-PET and MRI.

	AD, FTLD and Controls	AD vs FTLD	AD vs Controls	FTLD vs Controls
GMwhole-brain	72.9%	80.0%	88.2%	77.8%
WMwhole-brain	66.7%	74.3%	79.4%	77.8%
FDG-PETwhole-brain	81.3%	82.9%	94.1%	92.6%
GM/ FDG-PETwhole-brain	79.2%	82.9%	94.1%	88.9%
GM/WM/FDG-PETwhole-brain	77.1%	82.9%	91.2%	85.2%
GM + FDG-PETwhole-brain	81.3%	88.6%	91.2%	88.9%
GMROIs	56.3%	60.0%	82.4%	85.2%
FDG-PETROIs	75.0%	80.0%	94.1%	85.2%
GM/FDG-PETROIs	91.7%	94.3%	100.0%	92.6%

Accuracy represents the percentage of subjects correctly assigned to the correct condition. AD Alzheimer's disease, FDG-PET [18F]fluorodeoxyglucose positron emission tomography, FTLD frontotemporal lobar degeneration, GM gray matter, MRI magnetic resonance imaging, ROI region-of-interest, SVM support vector machine, WM white matter.

### SVM – ROI analysis

Accuracy based on ROIs from both meta-analyses using only GM information was substantially lower for differentiation between AD and FTLD patients in comparison with whole-brain classification. However, it was comparable to the whole-brain approach in differentiating between patients with both types of dementia and control subjects. ROIs extracted from FDG-PET data showed slightly lower discrimination accuracy compared to whole-brain information. The best accuracy rates of all SVM classifications were obtained using combined information extracted from FDG-PET and GM data. This approach resulted in a classification accuracy of 92% for the differentiation of all three groups and an accuracy rate of 94% for differentiation between AD and FTLD patients. Sensitivity of this ROI-based classification ranged between 85.7% for FTLD and 100% for AD and specificity of 100% for discrimination of both types of dementia from control subjects (Table 4).

**Table 4:** Differentiation rates for combined region-of-interest information from FDG-PET and MRI.

	Accuracy	Sensitivity	Specificity
AD vs FTLD	94.3%	95.2%*	92.9%
AD vs Controls	100.0%	100.0%	100.0%
FTLD vs Controls	92.6%	85.7%	100.0%

*Considering a correctly identified AD as a true positive. AD Alzheimer's disease, FDG-PET [18F]fluorodeoxyglucose positron emission tomography, FTLD frontotemporal lobar degeneration, MRI magnetic resonance imaging.

The classification accuracy in the 30 trials randomly assigning all subjects to the three groups resulted in a mean accuracy rate of 34±7.7% (mean ± standard deviation), ranging between 21 and 52% for the ROI-based SVM classification, and 33.7±8.2% ranging between 12 and 50% for the whole-brain classification.

## Discussion

In this study we performed a multimodal comparison and discrimination of dementia patients using FDG-PET and MRI. To enable a more accurate coevaluation of both imaging modalities, we developed a new preprocessing algorithm. This algorithm was designed to enable an accurate anatomical registration of both modalities. All processing steps were performed as far as possible simultaneously by applying the same deformations and preprocessing parameters to both modalities of the same subject. This procedure resulted in an accurate anatomical overlap of both imaging modalities and in an accurate between-subject registration, with both images having the same voxel size and approximately the same effective smoothness.

### SVM

SVM classification is a very promising tool for detection and differentiation of different dementia syndromes, as has been shown by previous studies [10],[12],[13],[24]. It not only captures univariate relationships of a single voxel across all subjects but is also able to detect multivariate relationships over a large group of information, as, for example, between different structures and modalities in the brain. Furthermore, this tool provides an easy way to use this information for classifying imaging data of new subjects to a specific condition.

Here, we systematically compared different information provided by FDG-PET and MRI to enable the most accurate detection and differentiation of dementia. The diagnosis was based on comprehensive clinical and neuropsychological testing. Although the data are not histopathologically confirmed to be sure of assigning them to the correct condition, generally higher conformity with the clinical diagnosis should also result in more accurate classification of histopathologically validated data.

The whole-brain SVM classification provided the most accurate classification using only FDG-PET information. GM and WM based classification accuracy was lower for all comparisons indicating a lower sensitivity for detection of dementia-relevant information. Nonetheless, classification based on GM, WM and FDG-PET separately or combining them revealed a discrimination accuracy which was above chance level for the correct categorization of the three groups. All classification results substantially exceeded the best classification accuracy obtained by randomly assigning all subjects to different groups. Additionally, smoothing of the data improved the classification accuracy in both imaging modalities as expected.

However, in whole-brain classification noise is introduced by using a great deal of information for classification that does not differentiate between the groups. Recent comprehensive meta-analyses identified the “prototypical” networks for both disorders in both modalities using VBM [8],[9]. The involved regions have been shown to be affected in AD and FTLD patients most consistently in all studies investigating these disorders. By using this information, we ruled out the possibility that our classification results are dependent to our group of patients. Although this method provides lower accuracy rates for GM or FDG-PET information on their own, it shows a significantly higher discrimination rate by combining both information modalities into a single vector. This ROI-based discrimination is superior to whole-brain classification with the highest accuracy gain for the differentiation of both types of dementia, which, with 94%, is the highest differentiation rate reported up to now. Accordingly, we suggest this method as a diagnostic standard for the classification of dementia syndromes.

Nonetheless, some limitations should be considered regarding the results of the present study. First of all, the number of subjects used for classification is too low to allow a generalizable conclusion for patients from other clinical centers. This is especially a problem because the accuracy of the clinical diagnosis for specific dementia syndromes, which was used here for validation, strongly varies between different clinical centers. Therefore, in a future work this approach should be validated using a larger and more generalizable dataset like the data provided by the Alzheimer's disease Neuroimaging Initiative (ADNI: www.adni-info.org). A further limitation of the present work is the significantly younger age of the control group in comparison to the patient cohort. However, this aspect might only have contributed to the discrimination between dementia patients and control subjects but not to the high classification accuracy of AD and FTLD patients as these were very similar in their age range. If age contributed to the classification accuracy there should be lower classification accuracy for young dementia patients and older control subjects as they did not differ in age. For comparison of both types of dementia patients and control subjects the classification accuracy did not differ for younger and older dementia patients although half of the patients were in the same age range as the control group. In AD group all patients were classified correctly. In FTLD group one younger and one older patient were misclassified. Independently of age, all control subjects were classified correctly for both comparisons. These results indicate that the slight mean age differences is not the decisive factor for the high discrimination accuracy using combined information from FDG-PET and MRI. Furthermore, if age still slightly contributed to the high discrimination of dementia patients and control subjects this contribution was also present in all other single modality and multimodal whole-brain and ROI-based SVM classifications applied in this study. Therefore, age cannot account for increased differentiation accuracies when combined ROI information from FDG-PET and MRI are used for differentiation of dementia patients and control subjects.

Another point is that subjects in the control group in our study reported subjective cognitive complaints which might have limited the interpretation of the results of our study. However, only subjects were included whose cognitive complaints were not confirmed by comprehensive neuropsychological evaluation. The CDR is a semi-structural interview and is highly dependent on the subjectively perceived memory impairment which resulted in a CDR score of 0.5 for these control subjects in our study. In recent literature it has been shown that the CDR stage of 0.5 has a poor discriminative value for healthy control subjects and subjects with Mild Cognitive Impairment (MCI) [42],[43]. Meguro et al. [42] have shown that about 30% of a normal population older then 65 got a CDR score of 0.5 while the prevalence of MCI in the same population was only about 5% which suggests that CDR score of 0.5 is not a good indicator of MCI. Due to the absence of any objective cognitive impairment in all neuropsychological tests for all subjects included in the control group in our study this group of subjects can be regarded as cognitively unimpaired.

### Conclusion and perspectives

In our study, we investigated the advantages of SVM classification using combined information from FDG-PET and MRI to improve detection and differentiation of dementia. Furthermore, based on affected regions reported in previous studies, investigating Alzheimer's disease and frontotemporal lobar degeneration with univariate approaches and summarized in two meta-analyses, we applied linear support vector machine classification algorithm using information from both imaging modalities. Combining region-of-interest information from FDG-PET and MRI resulted in a substantial gain in accuracy compared to whole-brain and to single modality classification for both detection and differentiation of Alzheimer's disease and frontotemporal lobar degeneration. Our results indicate that integration and combination of results from different imaging modalities might provide a new way to improve the diagnostic accuracy of these dementia disorders.
